# Microbial and metabolomic mechanisms mediating the effects of dietary inulin and cellulose supplementation on porcine oocyte and uterine development

**DOI:** 10.1186/s40104-021-00657-0

**Published:** 2022-01-13

**Authors:** Zhaoyue Men, Meng Cao, Yuechan Gong, Lun Hua, Ruihao Zhang, Xin Zhu, Lianchao Tang, Xuemei Jiang, Shengyu Xu, Jian Li, Lianqiang Che, Yan Lin, Bin Feng, Zhengfeng Fang, De Wu, Yong Zhuo

**Affiliations:** 1 Animal Nutrition Institute, Sichuan Agricultural University, 211 Huimin Road, Wenjiang District, Chengdu, 611130 People’s Republic of China; 2 College of Animal Science and Technology, Sichuan Agricultural University, Chengdu, 611130 People’s Republic of China

**Keywords:** Dietary fiber, Gilts, Metabolomics, Microbiota, Oocyte maturation

## Abstract

**Background:**

Dietary fiber (DF) is often eschewed in swine diet due to its anti-nutritional effects, but DF is attracting growing attention for its reproductive benefits. The objective of this study was to investigate the effects of DF intake level on oocyte maturation and uterine development, to determine the optimal DF intake for gilts, and gain microbial and metabolomic insight into the underlying mechanisms involved.

**Methods:**

Seventy-six Landrace × Yorkshire (LY) crossbred replacement gilts of similar age (92.6 ± 0.6 d; mean ± standard deviation [SD]) and body weight (BW, 33.8 ± 3.9 kg; mean ± SD) were randomly allocated to 4 dietary treatment groups (*n* = 19); a basal diet without extra DF intake (DF 1.0), and 3 dietary groups ingesting an extra 50% (DF 1.5), 75% (DF 1.75), and 100% (DF 2.0) dietary fiber mixture consisting of inulin and cellulose (1:4). Oocyte maturation and uterine development were assessed on 19 d of the 2nd oestrous cycle. Microbial diversity of faecal samples was analysed by high-throughput pyrosequencing (16S rRNA) and blood samples were subjected to untargeted metabolomics.

**Results:**

The rates of oocytes showing first polar bodies after in vitro maturation for 44 h and uterine development increased linearly with increasing DF intake; DF 1.75 gilts had a 19.8% faster oocyte maturation rate and a 48.9 cm longer uterus than DF 1.0 gilts (*P* <  0.05). Among the top 10 microbiota components at the phylum level, 8 increased linearly with increasing DF level, and the relative abundance of 30 of 53 microbiota components at the genus level (> 0.1%) increased linearly or quadratically with increasing DF intake. Untargeted metabolic analysis revealed significant changes in serum metabolites that were closely associated with microbiota, including serotonin, a gut-derived signal that stimulates oocyte maturation.

**Conclusions:**

The findings provide evidence of the benefits of increased DF intake by supplementing inulin and cellulose on oocyte maturation and uterine development in gilts, and new microbial and metabolomic insight into the mechanisms mediating the effects of DF on reproductive performance of replacement gilts.

**Supplementary Information:**

The online version contains supplementary material available at 10.1186/s40104-021-00657-0.

## Background

Dietary fiber (DF) is often excluded from animal feed due to its anti-nutritional properties during nutrient digestion in monogastric nutrition [1, 2]. However, DF reportedly benefits swine production, including improving the welfare of gestating sows fed a restricted diet [3]. In recent decades, the inclusion of DF in the diets of replacement gilts has received growing attention due to its beneficial effects on reproductive performance. Gilts fed a fiber-rich diet by adding high levels of sugar beet pulp 19 d prior to breeding displayed greater embryo survival (88.2%) at 28 d of pregnancy than controls (80.0%), while increasing the feeding level (from 1.8 × maintenance to 2.6 × maintenance), starch (+ 451 g/d) or protein (+ 158 g/d) did not improve embryonic survival [4]. Furthermore, this beneficial effect of high DF prior to mating on the survival of early embryos acted by improving the quality of oocytes [5]. However, feeding replacement gilts a lupin-based high-fiber diet, but not a wheat bran-based diet, accelerated oocyte maturation [6], adding complexity to the effects of DF on the reproductive outcomes of gilts. Most previous researches on the effects of fiber have explored high levels of fiber-rich ingredients such as sugar beet pulp. However, other nutrients (e.g., vitamins) complicate the direct effects of DF. Adding extracted forms of fiber such as inulin, cellulose and pectin allows the direct evaluation of the effect of DF [7–10]. Recently, we investigated the effects of different levels of DF intake on the ovarian follicle reserve of gilts [7], but the optimal level of dietary fiber for oocyte maturation and uterine development in gilts of mating age remains unknown.

As mentioned above, some of the beneficial effects of DF on reproductive performance have been elucidated, but the underlying mechanism remains largely uncertain. DF is usually mobilised by gut microbiota to generate short-chain fatty acids (SCFAs) such as acetate, propionate and butyrate [11]. Additionally, SCFAs can be taken up by peripheral tissues such as the stomach, intestine, liver, adipocytes and skeletal muscle, making it difficult for them to reach threshold concentrations to activate downstream targets [11, 12]. Peripheral tissues in turn detect metabolites and respond accordingly by secreting secondary metabolic hormones such as serotonin [13]. However, it remains unclear which metabolites or metabolic hormones are involved in controlling the reproductive functions of replacement gilts.

Indeed, DF is generally defined as a carbohydrate that is neither absorbed nor hydrolysable by mammalian endogenous digestive enzymes. Although DF is gradually considered as an essential nutrient for normal gastrointestinal tract physiology and overall health of both human and domestic animals, there have been different methods for the quantification of DF within feeds/foods for both animal and human nutrition [14, 15]. “Crude fiber” (CF) was one of the earliest parameters to describe the DF, and later the Van Soest method was introduced to classify the DF into neutral detergent fiber (NDF), acid detergent fiber (ADF), and acid detergent lignin (ADF) in animal nutrition [15]. More recently, a simple classification of DF was introduced with enzymatic-gravimetric method, which allows the categorization of DF into “soluble” or “insoluble” based on the ability to be fully dispersed with water [16]. Soluble fiber has generally a high affinity in the water, and is easily hydrolyzed by the carbohydrate-active enzymes secreted by the microbiota in the gut, whereas insoluble fiber was less fermentable [1]. Additionally, the DF benefits could be attributed to its different physical characteristics such as water-holding capacity, viscosity, absorptive capacity, and faecal bulking capacity, as well as chemical characteristic fermentability [17]. Insoluble fibers (e.g. cellulose) usually related to water-holding capacity, absorptive capacity, and faecal bulking capacity, while soluble fibers (e.g. inulin) usually contributed to viscosity and fermentability [1]. This leads us to hypothesize that a combination of both soluble and insoluble fiber could optimize the effects of DF. The objective of this study was to investigate the effects of different DF levels by supplementing inulin and cellulose to the diets of growing gilts on oocyte quality and uterine development. We also probed changes in microbial diversity, performed metabolomic profiling based on 16S rRNA analysis, and conducted untargeted metabolic pathway analysis.

## Materials and methods

This trial was conducted at the research centre of Sichuan Agricultural University. Procedures were performed in accordance with the National Research Council Guide for the Care and Use of Laboratory Animals, and followed the regulations of the Animal Care and Use Committee of Sichuan Agricultural University (Approval No. 20174310).

### Animals, diets and experimental design

**Fig. 1:**
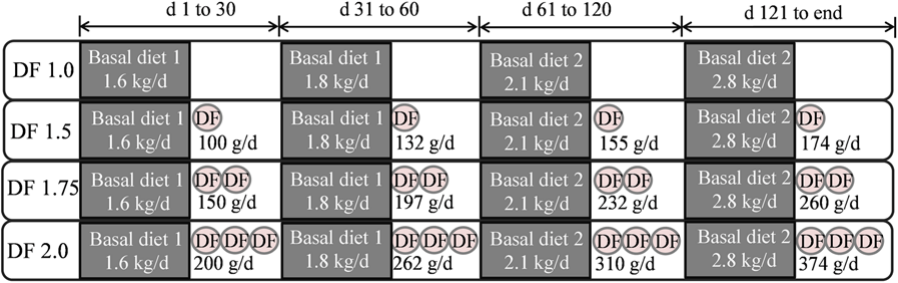
Schematic diagram of the experimental design. Two basal diets were formulated during 1 to 60 d and 61 d to the end of the experiment. Gilts were fed a basal diet, or a basal diet with three levels of extra dietary fiber (DF) during each phase. DF 1.0, basal diet without DF supplement, and DF 1.5, DF 1.75, and DF 2.0 were basal diets with an additional 50%, 75% and 100% DF, respectively. The DF mixture comprised inulin and cellulose at a ratio of 1:4

This was a companion trial of our recent study [7]. Seventy-six Landrace × Yorkshire (LY) crossbred replacement gilts of similar age (92.6 ± 0.6 d; mean ± SD) and body weight (33.8 ± 3.9 kg; mean ± SD) were used in this study. Gilts were randomly allocated to 4 dietary treatment groups (*n* = 19); a basal diet without extra DF intake (DF 1.0), and 3 dietary groups with 3 different levels of extra DF intake. Basal diets were divided into 2 phases; 1 to 60 d (72.0% corn, 20.8% soybean) and 61 d to the end of the experiment (78.0% corn, 16.0% soybean), respectively. The detailed diet formulation was shown in Table 1. The diet from 1 to 60 d of experiment contained 72.0% corn, 20.8% soybean meal, 2.5% fishmeal, and 2.0% soybean meal to provide 3.4 Mcal/kg of digestible energy, 16.9% of crude protein, and 1.08% of total lysine. The diet from 61 d to the end of experiment contained 78.0% corn, 16.0% soybean meal, 2.0% fishmeal, and 1.7% soybean meal to provide 3.4 Mcal/kg of digestible energy, 14.7% of crude protein, and 0.86% of total lysine. The soluble and insoluble fibers in basal diets were analysed by enzymatic-gravimetric method with minor modification [10]. Briefly, feed samples (1.0 g) were treated with a 40-mL MES-TRIS buffer solution (Sigma-Aldrich, Saint Louis, USA) on a stirrer. The heat-stable α-amylase solution (50 μL, A3306, Sigma-Aldrich) was added to the mixture and then incubated in a 95–100 °C water bath for 15 min with continuous agitation. The protease solution (P3910, Sigma-Aldrich) were then added for 30 min at 60 °C. Additional 300 μL amyloglucosidase solution (A9913, Sigma-Aldrich) was added to the solution for 30 min at 60 °C after adjusting pH to 4.0–4.7. After hydrolysis, the insoluble fiber residue was obtained by filtration on a crucible with acid washed wet and redistribute Celite (C8656, Sigma-Aldrich), and the filtrate was collected by adding 95% ethanol prewarmed at 60 °C to form the SDF precipitate. Total DF were calculated with sum of soluble fiber and insoluble fiber. The total DF in basal diets were 12.52% (d 1 to 60 of experiment) and 12.42% (d 61 to the end of experiment), respectively. DF 1.0 gilts were provided with 1.6, 2.1, 2.5 and 2.8 kg/d of basal diet and estimated total DF intake from diets was 200.3, 262.9, 310.5 and 347.8 g/d from 1 to 30 d, 31 to 60 d, 61 to 120 d, and 121 d to the end of the experiment, respectively. During each feeding phase, gilts were fed a basal diet supplemented with 50% (DF 1.5), 75% (DF 1.75) and 100% (DF 2.0) extra DF compared with gilts in the DF 1.0 group (Fig. 1). Equal amounts of feed were provided to gilts twice daily at 08:00 and 14:30 h. Extracted DF inulin and cellulose were composed of a 1:4 ratio, and this ratio was formulated as previously described [7, 8, 10]. All gilts were individually housed in a pen (2.0 m × 0.8 m) in a breeding facility with an environmental temperature maintained between 20 °C and 24 °C. Water was provided ad libitum. The onset of first puberty and the 2nd oestrous cycle were carefully checked in order to collect ovarian samples on 19 d of the 2nd oestrous cycle [7].

**Table 1:** Ingredients and nutrient compositions of basal diets (as fed basis), g/kg

Ingredients, g/kg	Phases of experiment	
	1–60 d	61 d–slaughter^3^
Corn	720	780
Soybean (44%CP)	208	160
Fish meal (65%CP)	25	20
Soybean oil	20	17
L-Lys HCl (98%)	3	2
DL-Met (99%)	1	0.4
L-Thr (98%)	0.6	0.2
L-Trp (98%)	0.1	0
Choline chloride (50%)	1.5	1.5
Sodium chloride (feed grade, > 99.0%)	4	4
Limestone	6.2	5.9
Monocalcium phosphate	8.6	7
Vitamin-mineral premix^1^	2	2
Total	1000	1000
Nutrient composition, g/kg		
Digestible energy, Mcal/kg	3.4	3.4
Crude protein	169.1	147.2
Total Lysine	10.8	8.6
SID Lysine	9.8	7.8
Calcium	6.9	5.9
Total phosphorus	5.9	5.3
Soluble fiber	10.2	10.3
Insoluble fiber	115.0	113.9
Total dietary fiber^2^	125.2	124.2

^1^Provided the following per kilogram of basal diet: 8000 IU vitamin A; 800 IU vitamin D_3_; 30 IU vitamin E; 4 mg vitamin K; 0.16 mg biotin; 2 mg folacin; 25 mg niacin; 20 mg pantothenic acid; 10 mg riboflavin; 2 mg thiamine; 1 mg vitamin B_6_; 20 μg vitamin B_12_; 16 mg copper as as copper sulfate; 0.25 mg iodine as potassium iodide; 125 mg iron as ferrous sulphate; 30 mg manganese as manganese sulfate; 0.25 mg selenium as sodium selenite; 125 mg zinc as zinc sulfate

^2^Total dietary fiber = soluble fiber + insoluble fiber, analyzed value according to method AOAC 991.43

^3^Gilts were slaughtered at the 19th day of 3rd estrous cycle

### Sample collection

Blood samples were collected from gilts at 2 h after the morning meal at both 30 d of the experiment and 19 d of the 2nd oestrous cycle. Blood samples were centrifuged at 3000 × *g* at 4 °C for 30 min to collect serum, and stored at − 20 °C for future analysis.

Faecal samples were randomly collected (*n* = 8 per group) at 30 d of the experiment and at 19 d of the 2nd oestrous cycle. Defecation was promoted by rectal stimulation and faeces were collected immediately, transferred into sterile tubes with a sterile cotton swab pre-wetted with ice-cold sterile phosphate-buffered saline (PBS, pH 7.2), immediately snap-frozen in liquid N_2_, and stored at − 80 °C. All contacts with faeces were kept sterile during the entire sampling procedure to avoid contamination.

Collection of reproductive organs was performed at 2 time points. At 30 d of the experiment, 24 gilts (6 gilts per group) were randomly chosen for harvesting bilateral ovaries 2 h after the morning meal under anaesthesia. Cumulus-oocyte complex (COC) and follicular fluid samples were also collected from antral follicles (diameter 1–3 mm) on the surface of ovaries, as previously described [18], snap-frozen, and stored at − 80 °C. At 19 d of the 2nd oestrous cycle, another 24 gilts (6 gilts per group) were slaughtered for collecting ovarian, oviduct and uterine samples. Ovaries were washed with PBS prewarmed at 39 °C, maintained at 39 °C in TCM199 medium (Gibco, USA) containing 0.1% polyvinyl alcohol (Sigma, USA), and transported to the laboratory within 1 h after sample collection. Uterus and oviduct samples were washed with ice-cooled PBS and dried with sterile tissue paper, and their weight and length in both directions (left and right) were measured.

Colonic contents at the proximal section were quickly transferred to 1.5-mL sterile tubes, washed 3 times with ice-cold PBS, and dried with sterile tissue paper. Both colonic tissues and contents were snap-frozen in N_2_ and stored at − 80 °C.

### Oocyte maturation in vitro

COCs collected from follicles with diameters between 3 mm and 6 mm at 19 d of the 2nd oestrous cycle were subjected to in vitro maturation to measure their oocyte quality as previously described with minor modifications [18]. In brief, COCs were aspirated from large follicles with a 10-mL syringe equipped with an 18-gauge needle on a sterile operating table. COCs were carefully collected under a stereo microscope (Olympus, Japan), and only those with uniform oocyte cytoplasm and at least 2 layers of cumulus cells were selected for culture and maturation in vitro. Follicular fluid was harvested by centrifuging at 3000 × *g* at 4 °C and stored at − 20 °C for future analysis. The in vitro maturation medium was based on TCM199 medium, which was supplemented with 0.1% polyvinyl alcohol (Sigma), 10% porcine follicular fluid (from COCs with a diameter ≥ 3 mm), 3.05 mmol/L D-glucose (Sigma), 0.91 mmol/L sodium pyruvate (Sigma), 1× Penicillin-Streptomycin solution (Sigma), 0.57 mmol/L Cysteine (Sigma), 15 U/mL LH (Prospec, Israel), 15 U/mL FSH (Prospec) and 10 ng/mL EGF (Prospec). Cumulus cell expansion was measured after 22 h of culture in vitro by determining the expansion of cumulus cells surrounding oocytes using the following scoring scheme: Score 0, no expansion of cumulus cells; Score 1, a slight expansion of the outer layer of cumulus cells; Score 2, expansion of the outer two-to-three layers of cumulus cells; Score 3, expansion of 50% of cumulus cells; Score 4, full expansion of cumulus cells. Finally, evaluation of cumulus expansion was calculated by the following equation: rate of cumulus expansion (%) =[ total scores of COCs per gilt / (the number of COCs × 4)] × 100%. Next, cumulus cells were removed from COCs by gentle vortexing in 0.1% hyaluronidase (Sigma) in TCM 199 after in vitro maturation for 44 h, and the maturation of oocytes to metaphase II (MII) at 44 h of culture was evaluated based on the presence of the first polar body as previously described [18, 19].

### Analysis of SCFAs and microbiota

Along with the colonic contents, levels of acetate, propionate and butyrate SCFAs in faecal samples at 30 d of the experiment and at 19 d of the 2nd oestrous cycle were determined using a Varian CP-3800 gas chromatograph (manual injection, flame ionisation detector, 10 μL microinjector; Varian), as previously described [13]. Microbial diversity in faeces at 19 d of the 2nd oestrous cycle was measured using high-throughput pyrosequencing (16S rRNA analysis). Detailed procedures were conducted as previously described [10], and they are presented in the online supplementary methods.

### Measurement of serotonin and melatonin

The concentrations of serotonin (DLD Diagnostika GmbH) and melatonin (IBL #RE54021) in serum and follicular fluid samples were measured with their respective ELISA kits as recently described [13]. Additionally, the serotonin content in proximal colon tissues was normalised to tissue weight.

### Gene expression

Gene expression in ovarian COCs was investigated by real-time PCR. In brief, RNA was extracted with TRIzol reagent (TaKaRa, Dalian, China) for the synthesis of cDNA using a commercial reverse transcription kit (TaKaRa) was used. A 7900HT Fast Real-Time PCR System (Thermo Fisher Scientific) with SYBR Green Real-Time PCR reagent (RR820A, TaKaRa) was used to measure mRNA levels. Primers for target genes were bone morphogenetic protein 15 (*BMP15*) forward 5′-AGCTTCCACCAACTGGGTTGG-3′ and reverse 5′-TCATCTGCATGTACAGGGCTG-3′, growth differentiation factor 9 (*GDF9*) forward 5′-GGTATGGCTCTCCGGTTCACAC-3′ and reverse 5′- CTTGGCAGGTACGCAGGATGG-3′, *β*-actin, forward 5′-GGCCGCACCACTGGCATTGTCAT and reverse 5′- AGGTCCAGACGCAGGATGGCG-3′. The threshold cycle (2^–ΔΔCt^) method was used to calculate relative gene expression. *β*-actin was used as the housekeeping gene, and relative gene expression levels are expressed as fold changes relative to those in the DF 1.0 group.

### Untargeted metabolomics

Sera from 8 gilts per group were used for untargeted metabolomics analysis. In each group, 2 serum samples were randomly pooled as one sample, resulting in 4 replicate samples for each group. The detailed procedures, including metabolite extraction, UHPLC-MS/MS analysis, database search, and data analysis are presented in the online supplementary materials.

### Statistical analysis

Raw data were checked using the Grubb’s test method. If |*Xp* - *X*| > *λ* (*α*, *n*) *S*, then *Xp* was considered an outlier. Measurement data were normally distributed after testing for homogeneity of variance and normal distribution using the Shapiro-Wilk method in SAS 9.4 (SAS Institute Inc., Cary, NC, USA). As a completely randomised design, the statistical analyses were performed through the mixed procedure of SAS 9.4 using the following statistical model: *Yij* = *μ* + *Ti* + *eij*, where *Y* is the analysed variable, *μ* is the overall mean, *Ti* is the fixed effect of the *i*th treatment, and *eij* is the error term specific to the pig identified assigned to the *i*th treatment. The linear and quadratic effects of increasing DF levels on the analysed variable were determined by orthogonal polynomial contrast. Differences were considered statistically significant when *P* <  0.05 and a trend was considered significant when 0.05 ≤ *P* <  0.10.

## Results

### Nutrient intake and number of gilts at each stage

Six gilts per group were removed from the experiment after collection of their ovaries at 30 d of the experiment, 13 gilts remained per group from 31 d of the experiment. Additionally, 9 gilts (4, 2, 2 and 1 in DF 1.5, DF 1.75 and DF 2.0, respectively) were excluded since they did not show oestrous at 240 days of age. In this study, gilts in each treatment group could consume their provided feed; therefore, gilts in each group were expected to consume similar levels of digestible energy, amino acids, minerals and vitamins, but with different levels of DF intake. The estimated average daily DF intake was 284.28 g/d, 420.92 g/d, 494.91 g/d and 568.16 g/d for DF 1.0, DF 1.5, DF 1.75 and DF 2.0 groups, respectively, throughout the experimental period. The average daily gain in body weight at 30 d of experiment and at 19 d of the 2nd oestrous cycle were reported in a companion study [7], and were not affected by dietary treatment.

### Effects of DF intake level on oocyte quality and reproductive organ development

**Fig. 2:**
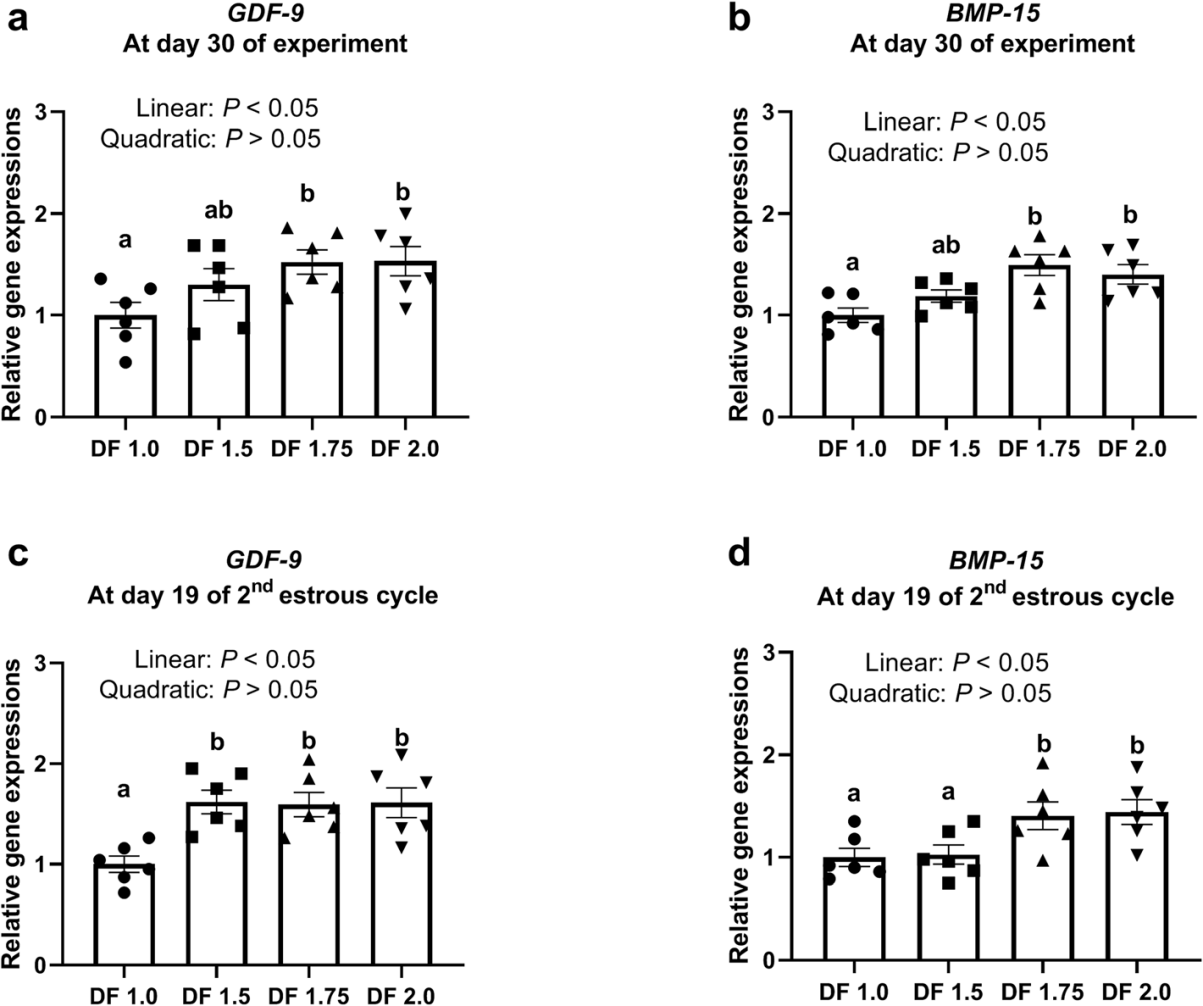
Effects of DF intake level on gene expression of COCs in gilts. *GDF*-9, Growth differentiation factor-9; *BMP-15*, Bone morphogenetic protein 15; DF 1.0, basal diet without DF supplement; DF 1.5, DF 1.75, and DF 2.0, basal diets with an additional 50, 75 and 100% DF intake, respectively. *n* = 6 per group. Columns with different letters ^a,b^ denote *P* < 0.05

As shown in Table 2, the number of COCs collected per gilt ranged between 21.2 and 23.3, and the number of oocytes used for in vitro maturation ranged between 15.5 and 15.7, and these were not affected by DF intake level (*P* > 0.05). The expansion rate of COCs was not affected by DF level (*P* > 0.05). The rate of oocytes with first polar bodies increased linearly with increasing DF level (*P* = 0.001), and was significantly higher for the DF 1.75 diet than for the DF 1.0 diet (57.5% vs. 37.7%, *P* <  0.05). The mRNA expression levels of *GDF-9* and *BMP-15*, two markers of oocyte quality in ovarian COCs of gilts (Fig. 2a-d), were increased linearly with increasing DF intake level at 30 d of the experiment and 19 d of the 2nd oestrous cycle.

**Table 2:** Effects of DF intake level on oocyte maturation in gilts

Items	Treatments				*P*-value	
	DF 1.0	DF 1.5	DF 1.75	DF 2.0	Linear	Quadratic
No. of COCs collected per gilt	23.3 ± 1.4	21.2 ± 1.2	22.2 ± 0.9	22.5 ± 2.3	0.695	0.403
No. of oocytes for in vitro maturation	15.5 ± 0.2	15.7 ± 0.2	15.7 ± 0.2	15.5 ± 0.2	0.898	0.467
Expansion rate, %	86.9 ± 5.9	89.5 ± 3.5	91.5 ± 2.1	93.6 ± 3.3	0.232	0.876
Rate of oocytes with first polar body, %	37.7 ± 3.2^c^	41.6 ± 3.0^bc^	57.5 ± 2.5^a^	50.6 ± 2.3^ab^	0.001	0.568

Data are expressed as means ± standard error (S.E.); *DF*, dietary fiber; *n* = 6; Means with different letters ^a,b,c^ denote *P* <  0.05

The effects of DF intake level on the development of reproductive organs (uterus and oviduct) are shown in Table 3. The weight of the uterus (*P* = 0.059) and the relative weight of the uterus (*P* = 0.017) increased linearly with increasing DF intake level. The relative weight of the uterus in gilts increased from 5.44 g/kg BW in the DF 1.0 group to 6.21 g/kg BW in the DF 1.75 group (*P* <  0.05). The lengths of the left uterine horn (*P* = 0.044) and the right uterine horn (*P* = 0.001) were increased by DF intake level. Specifically, DF 1.75 gilts had a 19.8% greater oocyte maturation rate and a 48.9 cm longer uterus length than DF 1.0 gilts (*P* <  0.05). The weight of the left oviduct (*P* = 0.087) and the right oviduct (*P* = 0.002) increased linearly with increasing DF intake level.

**Table 3:** Effects of DF intake level on the development of reproductive tracts in gilts

Items	Treatments				*P*-value	
	DF 1.0	DF 1.5	DF 1.75	DF 2.0	Linear	Quadratic
BW at laughter, kg	146.7 ± 1.6	145.3 ± 1.8	145.8 ± 3.0	142.3 ± 1.7	0.209	0.549
Weight of uterus, kg	0.80 ± 0.02	0.82 ± 0.02	0.90 ± 0.03	0.84 ± 0.02	0.059	0.380
Relative weight of uterus, g/kg	5.44 ± 0.17^b^	5.64 ± 0.16^ab^	6.21 ± 0.22^a^	5.91 ± 0.14^ab^	0.017	0.548
Left uterine, cm	99.2 ± 4.3^b^	107.3 ± 3.3^ab^	118.2 ± 5.3^a^	109.2 ± 5.3^ab^	0.044	0.232
Right uterine, cm	91.8 ± 4.2^c^	99.3 ± 3.1^bc^	121.7 ± 6.3^a^	113.5 ± 6.0^ab^	0.001	0.660
Left oviduct, g	3.93 ± 0.25	4.02 ± 0.47	5.19 ± 0.36	4.64 ± 0.50	0.087	0.880
Right oviduct, g	3.63 ± 0.22^b^	4.12 ± 0.54^b^	5.91 ± 0.30^a^	4.97 ± 0.38^ab^	0.002	0.446
Left oviduct, cm	30.5 ± 0.9	33.3 ± 2.6	35.3 ± 1.0	33.8 ± 1.7	0.100	0.400
Right oviduct, cm	30.6 ± 1.1	32.7 ± 2.5	36.5 ± 1.2	33.2 ± 2.1	0.123	0.343

Data are expressed as means ± S.E.; *DF*, dietary fiber; *n* = 6; Means with different letters ^a,b^ denote *P* <  0.05

### Effects of DF intake level on faecal microbial diversity at 19 d of the 2nd oestrous cycle

**Fig. 3:**
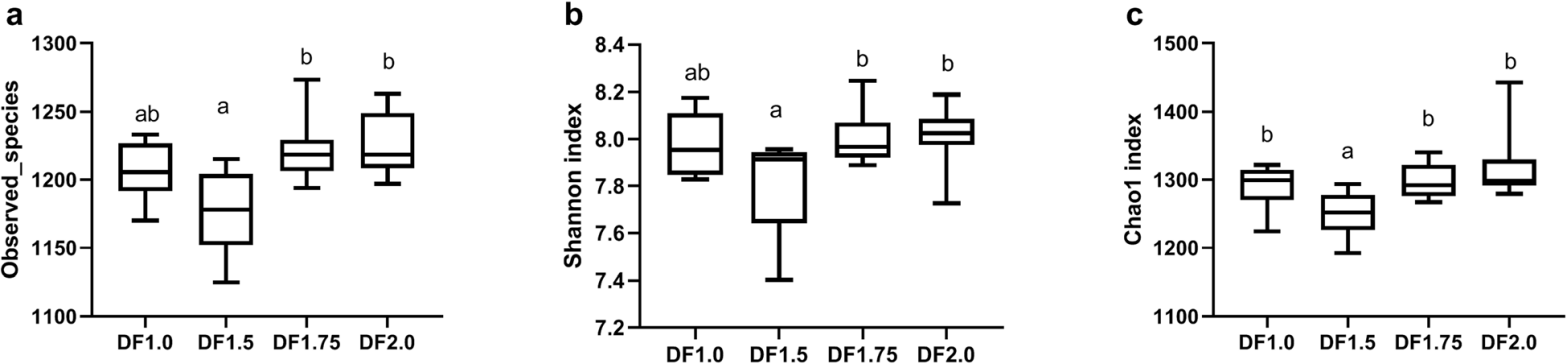
Microbiota alpha-diversity in faeces of gilts fed different dietary fiber levels at 19 d of the 2nd oestrous cycle. a, Observed species. b, Shannon index. c, Chao 1 index. Gilts constituted the experimental units (*n* = 6). DF 1.0, basal diet without DF supplement; DF 1.5, DF 1.75 and DF 2.0, basal diets with an additional 50%, 75% and 100% DF intake, respectively. Columns with different letters ^a,b^ denote *P* < 0.05

Total tags, unique tags, taxon tags, and operational taxonomic units (OTUs) of faecal microbiota at 19 d of the 2nd oestrous cycle in the 4 dietary groups were 1,721,847, 242,996, 1,478,851 and 31,092, respectively, at the 97% identity level, revealed by 16S rRNA sequencing. Microbiota alpha diversity was reflected by observed species, Shannon and Chao 1 indices. The observed species and Shannon indices were similar for DF 1.5 and DF 1.0 groups, but were lower than those of the DF 1.75 and DF 2.0 groups (Fig. 3a and b, *P* < 0.05). The Chao 1 index for the DF 1.5 group was lower than for the DF 1.0, DF 1.75 and DF 2.0 groups (Fig. 3c, *P* < 0.05).

As shown in the heatmap in Supplementary Fig. 1a and b, we identified clear differences in the phylum and genus distributions of faecal microbiota with increasing DF intake level. The relative abundances of microbiota at the phylum level in faeces of gilts at 19 d of the 2nd oestrous cycle are presented in Table 4. Two dominant phyla, Firmicutes and Bacteroidetes, accounted for ~ 85% of faecal microbiota. The relative abundance of the Firmicutes phylum decreased linearly (*P* <  0.001) or quadratically (*P =* 0.003) with increasing DF intake level. By contrast, the relative abundance of the Bacteroidetes phylum increased linearly (*P* <  0.001) or quadratically (*P =* 0.043) by increasing DF intake level. The relative abundance of the Proteobacteria phylum decreased linearly with increasing DF intake level (*P* = 0.002). The relative abundance of Tenericutes increased linearly with increasing DF intake level (*P* = 0.002). The relative abundance of the Actinobacteria and Planctomycetes phyla decreased linearly (*P* <  0.001) or quadratically (*P <* 0.05) with increasing DF intake level. The relative abundance of Cyanobacteria increased linearly (*P* <  0.001) or quadratically (*P =* 0.01) with increasing DF intake level.

**Table 4:** Relative abundance of microbiota at the phyla level in faeces of gilts, %

Items	Treatments				*P*-value	
	1.0 DF	1.5 DF	1.75DF	2.0 DF	Linear	Quadratic
Firmicutes	44.22 ± 1.07^ab^	47.63 ± 1.89^a^	40.34 ± 1.53^bc^	38.36 ± 0.80^c^	< 0.001	0.003
Bacteroidetes	40.64 ± 1.16^bc^	38.81 ± 1.79^c^	44.85 ± 1.78^ab^	46.51 ± 1.42^a^	0.004	0.043
Spirochaetes	4.62 ± 0.65^ab^	3.29 ± 0.59^b^	5.76 ± 0.63^a^	5.60 ± 0.79^ab^	0.169	0.079
Proteobacteria	4.08 ± 0.41^a^	3.80 ± 0.26^ab^	3.00 ± 0.23^b^	3.00 ± 0.24^b^	0.002	0.629
Tenericutes	2.12 ± 0.13	2.26 ± 0.27	2.73 ± 0.22	2.69 ± 0.16	0.013	0.866
Euryarchaeota	2.15 ± 0.32	2.72 ± 0.79	1.38 ± 0.38	1.97 ± 0.29	0.385	0.778
Actinobacteria	0.87 ± 0.55^a^	0.22 ± 0.01^b^	0.18 ± 0.02^b^	0.23 ± 0.03^b^	< 0.001	0.008
Lentisphaerae	0.74 ± 0.05^b^	0.67 ± 0.07^b^	1.02 ± 0.08^a^	0.82 ± 0.07^ab^	0.062	0.840
Planctomycetes	0.13 ± 0.04^ab^	0.26 ± 0.09^a^	0.09 ± 0.02^b^	0.05 ± 0.01^b^	0.017	0.002
Cyanobacteria	0.17 ± 0.02^bc^	0.15 ± 0.02^c^	0.25 ± 0.04^ab^	0.34 ± 0.05^a^	< 0.001	0.010
Others	0.25 ± 0.03^b^	0.18 ± 0.01^c^	0.41 ± 0.02^a^	0.47 ± 0.05^a^	< 0.001	< 0.001

Data are expressed as means ± S.E.; *DF*, dietary fiber; *n* = 6; Means with different letters ^a,b^ denote *P* <  0.05

The relative abundances of microbiota at the genus level (> 0.1%) are presented in Table 5. Thirty of the 53 genera increased linearly or quadratically changed with increasing DF intake level (*P* <  0.05 or *P* <  0.01). The relative abundances of the genera *Lactobacillus*, *Prevotella_9*, *Rikenellaceae_RC9_gut_group*, *Alloprevotella*, *Prevotellaceae_UCG-003*, *Prevotella_7*, *dgA-11_gut_group*, *Sphaerochaeta*, *Leeia*, *Erysipelotrichaceae_UCG-004*, *Catenibacterium*, *Fibrobacter* and *Faecalibacterium* were elevated by increasing DF intake level (linear or quadratic, *P* <  0.05). The relative abundances of the genera *Streptococcus*, *Clostridium_sensu_stricto_1*, *Succinivibrio*, *Eubacterium_coprostanoligenes_group*, *Ruminococcaceae_NK4A214_group*, *Lachnospiraceae_XPB1014_group*, *Phascolarctobacterium*, *Escherichia-Shigella*, *Family_XIII_AD3011_group*, *Turicibacter*, *Lachnospiraceae_AC2044_group*, *Candidatus_Soleaferrea*, *Lachnospira*, *Blautia*, *Acidaminococcus*, *Romboutsia*, and *Lachnoclostridium* decreased with increasing DF intake level (linear or quadratic, *P* <  0.05).

**Table 5:** Relative abundance of the top 53 microbiota at the genus level^1^,%

Items	Treatments				*P*-value	
	1.0 DF	1.5 DF	1.75 DF	2.0 DF	Linear	Quadratic
*Prevotellaceae_NK3B31_group*	6.33 ± 0.37	6.70 ± 0.80	6.34 ± 0.65	7.10 ± 1.04	0.440	0.895
*Lactobacillus*	2.21 ± 0.16^c^	2.60 ± 0.46^bc^	4.98 ± 1.10^a^	3.81 ± 0.32^ab^	< 0.001	0.660
*Prevotella_9*	3.46 ± 0.54^ab^	2.52 ± 0.30^b^	4.77 ± 0.73^a^	5.14 ± 0.92^a^	0.025	0.034
*Treponema_2*	4.23 ± 0.63	2.98 ± 0.57	4.94 ± 0.58	4.78 ± 0.79	0.368	0.119
*Rikenellaceae_RC9_gut_group*	4.33 ± 0.31^bc^	3.95 ± 0.33^c^	5.61 ± 0.34^ab^	5.80 ± 0.40^a^	0.001	0.045
*Streptococcus*	3.83 ± 0.28^b^	5.90 ± 0.56^a^	3.50 ± 0.50^b^	2.87 ± 0.29^b^	0.027	< 0.001
*Methanobrevibacter*	2.05 ± 0.32	2.66 ± 0.79	1.34 ± 0.38	1.89 ± 0.28	0.413	0.726
*Ruminococcaceae_UCG-005*	2.61 ± 0.27	3.16 ± 0.37	2.17 ± 0.23	2.15 ± 0.21	0.069	0.079
*Clostridium_sensu_stricto_1*	2.46 ± 0.29^a^	2.68 ± 0.16^a^	1.85 ± 0.10^b^	1.49 ± 0.11^b^	< 0.001	0.001
*Parabacteroides*	2.32 ± 0.26	2.24 ± 0.26	2.27 ± 0.24	2.15 ± 0.18	0.620	0.895
*Succinivibrio*	1.98 ± 0.30^a^	1.61 ± 0.17^ab^	1.00 ± 0.12^c^	1.11 ± 0.15^bc^	< 0.001	0.845
*Megasphaera*	1.88 ± 0.36	1.85 ± 0.69	0.87 ± 0.17	1.30 ± 0.50	0.089	0.838
*Ruminococcaceae_UCG-002*	2.42 ± 0.21	2.36 ± 0.21	2.30 ± 0.12	2.46 ± 0.24	0.989	0.571
*Eubacterium_coprostanoligenes_group*	2.06 ± 0.08^a^	2.42 ± 0.22^a^	1.58 ± 0.15^b^	1.51 ± 0.08^b^	0.001	0.001
*Prevotellaceae_UCG-001*	1.54 ± 0.37	1.24 ± 0.22	1.27 ± 0.34	1.67 ± 0.38	0.934	0.212
*Ruminococcaceae_NK4A214_group*	1.43 ± 0.08^ab^	1.50 ± 0.12^a^	1.18 ± 0.10^b^	1.16 ± 0.06^b^	0.008	0.169
*Oscillospira*	1.42 ± 0.08	1.32 ± 0.09	1.42 ± 0.14	1.41 ± 0.07	0.944	0.532
*Ruminococcaceae_UCG-014*	1.36 ± 0.14	1.31 ± 0.12	1.12 ± 0.12	1.18 ± 0.11	0.149	0.922
*Alloprevotella*	1.34 ± 0.13	1.28 ± 0.24	1.69 ± 0.14	1.92 ± 0.20	0.027	0.188
*Prevotellaceae_UCG-003*	1.28 ± 0.09^bc^	1.25 ± 0.20^c^	1.97 ± 0.15^a^	1.88 ± 0.17^ab^	0.002	0.382
*Lachnospiraceae_XPB1014_group*	1.12 ± 0.11	1.13 ± 0.08	0.94 ± 0.08	0.85 ± 0.06	0.011	0.191
*Prevotella_1*	1.00 ± 0.09	0.77 ± 0.10	0.79 ± 0.14	0.96 ± 0.14	0.680	0.095
*Ruminococcus_1*	0.98 ± 0.08	1.03 ± 0.16	0.82 ± 0.09	0.88 ± 0.10	0.271	0.710
*Phascolarctobacterium*	0.87 ± 0.09^a^	1.10 ± 0.16^a^	0.60 ± 0.06^b^	0.57 ± 0.03^b^	< 0.001	0.010
*Prevotella_2*	0.81 ± 0.08	0.66 ± 0.10	1.01 ± 0.12	1.04 ± 0.09	0.055	0.080
*Ruminococcaceae_UCG-010*	0.71 ± 0.04	0.67 ± 0.08	0.76 ± 0.03	0.81 ± 0.05	0.147	0.256
*Prevotella_7*	0.66 ± 0.13	0.36 ± 0.06	0.78 ± 0.15	0.89 ± 0.21	0.154	0.010
*Christensenellaceae_R-7_group*	0.60 ± 0.06	0.68 ± 0.06	0.50 ± 0.04	0.52 ± 0.04	0.093	0.168
*Escherichia-Shigella*	0.56 ± 0.22^a^	0.37 ± 0.09^ab^	0.15 ± 0.02^c^	0.23 ± 0.05^bc^	< 0.001	0.304
*dgA-11_gut_group*	0.52 ± 0.08	0.52 ± 0.07	0.68 ± 0.06	0.82 ± 0.12	0.008	0.179
*Family_XIII_AD3011_group*	0.50 ± 0.03^a^	0.48 ± 0.05^ab^	0.39 ± 0.02^b^	0.43 ± 0.02^ab^	0.018	0.734
*Terrisporobacter*	0.53 ± 0.05	0.52 ± 0.04	0.67 ± 0.08	0.53 ± 0.04	0.541	0.492
*Sphaerochaeta*	0.39 ± 0.03^b^	0.31 ± 0.03^b^	0.76 ± 0.10^a^	0.79 ± 0.04^a^	< 0.001	0.001
*Turicibacter*	0.33 ± 0.06^a^	0.33 ± 0.02^a^	0.23 ± 0.02^ab^	0.21 ± 0.02^b^	0.001	0.212
*Lachnospiraceae_AC2044_group*	0.34 ± 0.02^a^	0.29 ± 0.01^a^	0.18 ± 0.02^b^	0.19 ± 0.01^b^	< 0.001	0.764
*Leeia*	0.23 ± 0.04^b^	0.21 ± 0.08^b^	0.68 ± 0.27^a^	0.51 ± 0.08^ab^	0.002	0.534
*Mitsuokella*	0.25 ± 0.04	0.28 ± 0.09	0.33 ± 0.13	0.25 ± 0.04	0.794	0.368
*Candidatus_Soleaferrea*	0.22 ± 0.01^a^	0.25 ± 0.03^a^	0.13 ± 0.01^b^	0.16 ± 0.01^b^	< 0.001	0.207
*Lachnospira*	0.20 ± 0.03^a^	0.19 ± 0.01^b^	0.14 ± 0.01^b^	0.14 ± 0.01^b^	< 0.001	0.404
*Blautia*	0.21 ± 0.11^a^	0.10 ± 0.01^ab^	0.09 ± 0.02^b^	0.07 ± 0.01^b^	< 0.001	0.526
*Acidaminococcus*	0.18 ± 0.05^a^	0.17 ± 0.08^a^	0.04 ± 0.01^b^	0.08 ± 0.03^ab^	0.003	0.896
*Ruminococcaceae_UCG-009*	0.16 ± 0.01	0.15 ± 0.02	0.18 ± 0.02	0.17 ± 0.01	0.444	0.511
*Romboutsia*	0.15 ± 0.03^a^	0.14 ± 0.01^a^	0.08 ± 0.01^b^	0.08 ± 0.01^b^	< 0.001	0.366
*Erysipelotrichaceae_UCG-004*	0.14 ± 0.02^ab^	0.12 ± 0.01^b^	0.18 ± 0.02^ab^	0.19 ± 0.02^a^	0.024	0.064
*Catenibacterium*	0.14 ± 0.03^a^	0.05 ± 0.01^b^	0.19 ± 0.06^a^	0.25 ± 0.06^a^	0.019	< 0.001
*Bifidobacterium*	0.13 ± 0.01	0.11 ± 0.01	0.09 ± 0.02	0.14 ± 0.03	0.904	0.107
*Dialister*	0.12 ± 0.05	0.08 ± 0.02	0.20 ± 0.07	0.21 ± 0.08	0.061	0.058
*Fibrobacter*	0.12 ± 0.02	0.06 ± 0.01	0.18 ± 0.02	0.25 ± 0.05	< 0.001	< 0.001
*Lachnoclostridium*	0.12 ± 0.02	0.12 ± 0.02	0.07 ± 0.01	0.06 ± 0.01	< 0.001	0.075
*Thalassospira*	0.10 ± 0.01	0.20 ± 0.12	0.13 ± 0.01	0.12 ± 0.01	0.571	0.044
*Acetitomaculum*	0.10 ± 0.02	0.09 ± 0.02	0.12 ± 0.02	0.13 ± 0.03	0.178	0.457
*Campylobacter*	0.08 ± 0.01	0.11 ± 0.04	0.09 ± 0.02	0.10 ± 0.02	0.751	0.595
*Faecalibacterium*	0.08 ± 0.01^bc^	0.07 ± 0.01^c^	0.15 ± 0.02^a^	0.12 ± 0.01^ab^	0.004	0.475

Data are expressed as means ± S.E.; *DF*, dietary fiber; *n* = 6; Means with different letters ^a,b^ denote *P* <  0.05

*Effects of DF intake level on serum metabolomics at 19 d of the 2nd oestrous cycle.*

**Fig. 4:**
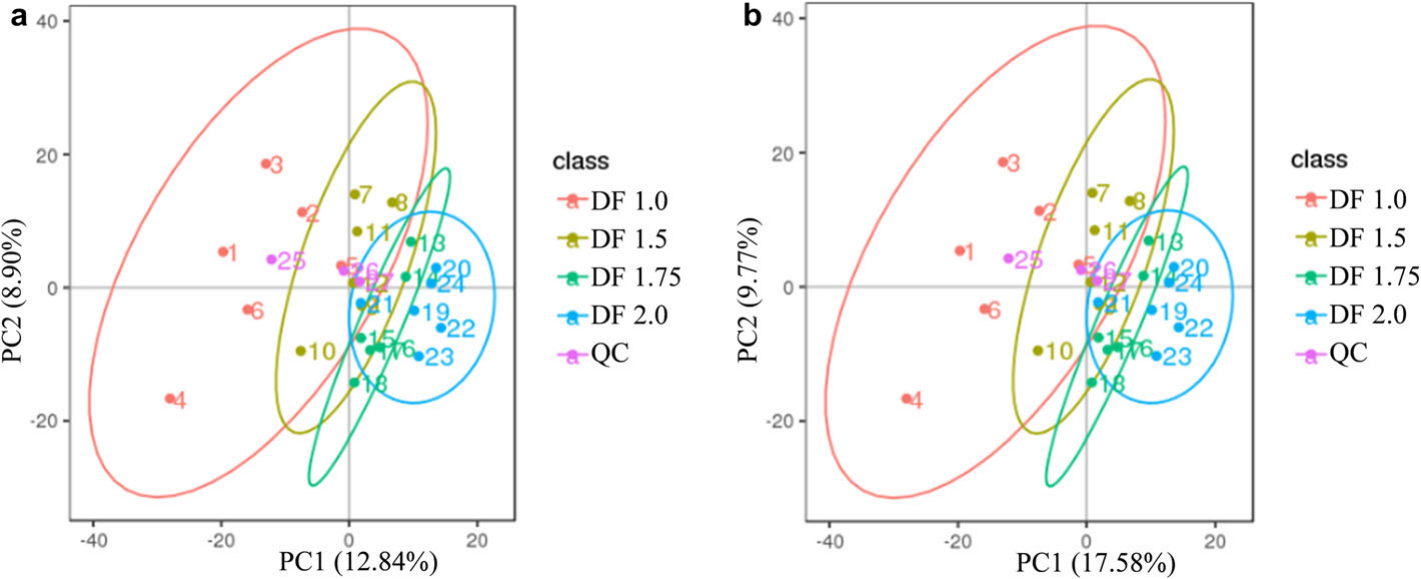
Principal component analysis of the metabolites identified in positive and negative ionisation modes. DF 1.0, basal diet without DF supplement; DF 1.5, DF 1.75 and DF 2.0, basal diets with an additional 50%, 75% and 100% DF intake, respectively. QC, quality control samples

As presented in Fig. 4, the principal component analysis (PCA) score plot of serum metabolomics data from both positive (a) and negative (b) ionisation modes showed a clear separation of metabolite communities between gilts in DF 1.0 and other groups, and differentially altered metabolites revealed significant changes in hierarchical clustering (Supplementary Fig. 2a and b). The numbers of differentially abundant metabolites identified and annotated in serum samples between groups are presented in Supplementary Table 1, revealing 92, 123 and 171 differentially altered metabolites in DF 1.5, DF 1.75 and DF 2.0 gilts compared with DF 1.0 gilts.

**Fig. 5:**
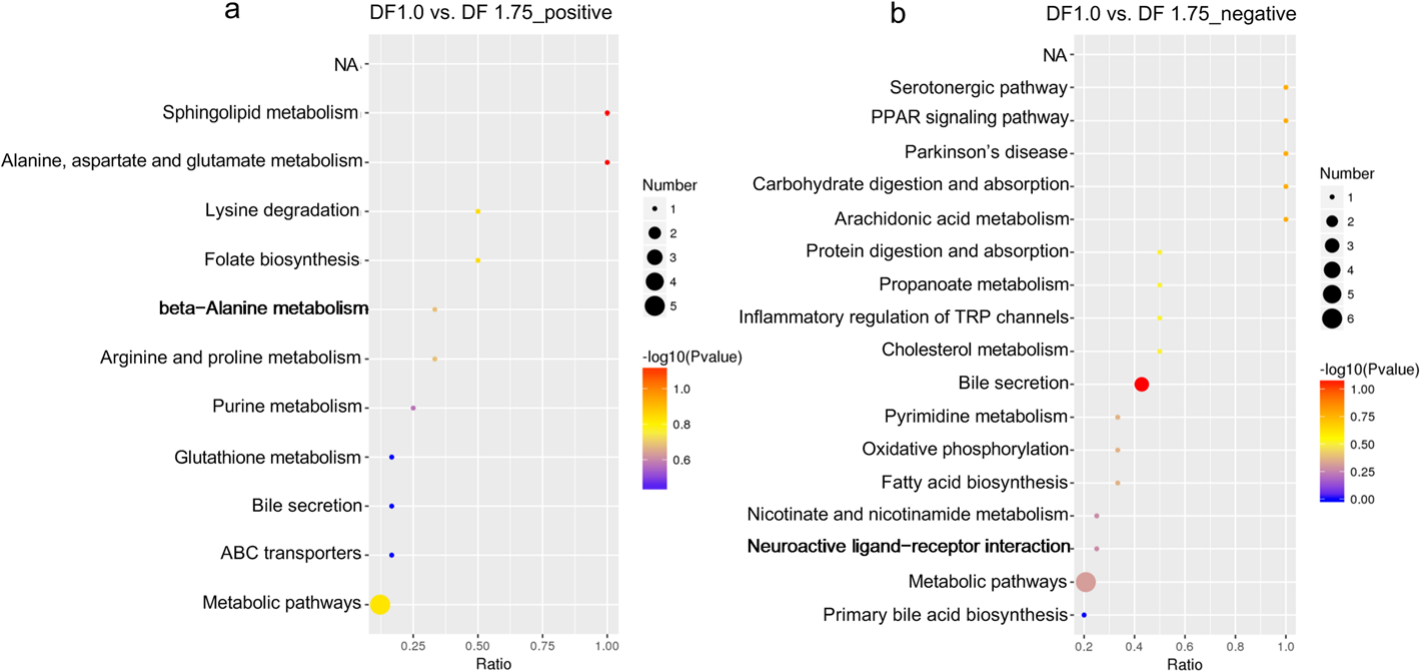
KEGG pathway analysis of differential metabolites identified in positive and negative ionisation modes between DF 1.0 and DF 1.75 groups. DF 1.0, basal diet without DF supplement; DF 1.75, basal diet with an additional 75% DF intake. DF, dietary fiber

In particular, we compared differentially abundant serum metabolites identified in both positive and negative ionisation modes between DF 1.0 and DF 1.75 gilts (Table 6). In brief, a total of 41 (in positive ionisation mode) and 20 (in negative ionisation mode) serum metabolites were upregulated by 1.5–27.0 times in DF 1.75 gilts compared with DF 1.0 gilts (*P* <  0.05 or *P* <  0.01). A total of 25 (in positive ionisation mode) and 36 (in negative ionisation mode) serum metabolites were down-regulated in DF 1.75 gilts compared with DF 1.0 gilts (*P* <  0.05 or *P* <  0.01). Enrichment of these metabolites resulted in changes in multiple biological pathways (Fig. 5), including the serotonergic pathway, the PPAR signalling pathway, Parkinson’s disease, carbohydrate digestion and absorption, arachidonic acid metabolism, protein digestion and absorption, propanoate metabolism, inflammatory mediator regulation of TRP channels, cholesterol metabolism, bile secretion, pyrimidine metabolism, oxidative phosphorylation, fatty acid biosynthesis, nicotinate and nicotinamide metabolism, neuroactive ligand-receptor interaction, and metabolic pathways under positive ionisation mode (Fig. 5a), and sphingolipid metabolism, alanine, aspartate and glutamate metabolism, lysine degradation, folate biosynthesis, metabolic pathways, arginine and proline metabolism, beta-alanine metabolism, purine metabolism, glutathione metabolism, ABC transporters, and bile secretion under negative ionisation mode (Fig. 5b).

**Table 6:** Differentially abundant serum metabolites between DF 1.0 and DF 1.75 gilts identified in positive and negative ionisation modes^1,2^

Name	Fold change	*P*-value	Name	Fold change	*P*-value
Positive ionisation			Negative ionisation		
(2S)-1-Hydroxy-3-(pentadecanoyloxy)- 2-propanyl (15Z)-15-tetracosenoate	18.966	0.047	Sinapyl alcohol	27.041	0.044
3-(2,4-Cyclopentadien-1-ylidene)- 5alpha-androstan-17beta-ol	5.594	0.003	Cuauhtemone	13.210	< 0.001
Oxandrolone	5.563	0.013	Cholic acid glucuronide	8.250	0.025
2,6-Di-tert-butyl-1,4-benzoquinone	5.028	0.005	GLIMEPIRIDE, CIS-	5.853	< 0.001
19-Nortestosterone	4.993	0.007	Olivoretin D	5.543	0.013
3-Beta-fluoro-5-beta-pregnan-20-one	4.670	0.002	Deoxycholic acid	4.117	0.001
p-Cymene	4.148	0.008	Avasimibe	3.204	0.026
Geroquinol	3.979	0.006	Taurochenodeoxycholic acid	3.100	0.044
Hypaphorine	3.902	< 0.001	p-Dimethylinamyl benzoate	3.077	< 0.001
Jasmonal	3.766	0.007	Chaksine	3.026	0.003
Ibuprofen	3.680	0.002	Tenivastatin	2.962	0.009
3-Beta,17-beta-diacetoxy-5α-androstane	3.057	0.007	Mupirocin	2.918	0.047
Oleandomycin 2′-O-phosphate	2.998	0.041	Ubiquinone Q4	2.219	0.025
Ionene	2.869	0.049	Sunitinib	2.185	0.011
Diaziquone	2.858	< 0.001	(3alpha)-3-Hydroxycholan-24-oic acid	2.054	0.042
1-Piperideine	2.857	0.004	Ifetroban	2.038	0.010
Myxalamid A	2.801	0.045	Maleimide	1.918	0.012
Genistein	2.584	0.011	5-HT	1.855	0.003
7-Ketodeoxycholic acid	2.434	0.023	Uldazepam	1.814	0.018
1-O-[4-(1H-indol-3-yl)butanoyl] -beta-D-glucopyranose	2.335	0.001	Indole-3-carboxilic acid-O-sulphate	1.690	0.019
Linagliptin	2.278	0.017	6α-Prostaglandin I1	0.566	0.011
(KDO)2-lipid IVA	2.096	0.045	3-Hydroxydecanoic acid	0.548	0.021
4-Aminobenzoic acid	2.089	0.043	14,18-Dihydroxy-12-oxo-9,13,15-octadecatrienoic acid	0.542	< 0.001
Spermidine	1.964	0.026	Cromoglicic acid	0.531	< 0.001
Hexoprenaline	1.902	0.005	Nemonapride (JAN)	0.526	0.044
Manumycin	1.899	0.041	Epithienamycin F	0.519	0.010
N-Lactoyl ethanolamine phosphate	1.888	0.002	10-Undecenoic acid	0.508	0.010
Perphenazine enantate	1.862	0.003	Prunin	0.502	0.010
5-HIAA	1.856	0.002	Leucodelphinidin	0.497	0.001
TU4153400	1.831	0.036	MFCD00065806	0.496	0.015
1-Octadecanoyl-2-[(15Z)- tetracosenoyl]-sn-glycero-3-phosphocholine	1.855	0.044	4,6,8-Trihydroxy-7-methoxy-3-methyl-3,4-dihydroisochromen-1-one	0.494	0.029
Bikhaconitine	1.850	0.007	ARAMITE	0.485	0.024
Cediranib	1.797	0.002	Uridine	0.480	0.013
Callystatin A	1.782	0.017	N-Palmitoyl-L-phenylalanine	0.463	0.014
Decoside	1.690	0.005	Cidofovir anhydrous	0.460	0.004
5-methyltetrahydropteroyltri- L-Glutamic acid	1.677	0.012	3-Indoxyl sulphate	0.448	0.002
2-(2-Carboxyethyl)-4-methyl- 5-Pentyl-3-furoic acid	1.640	0.012	Butoctamide semisuccinate	0.447	0.009
Trans-Anethole	1.637	0.021	Kukoamine A	0.443	0.001
Saccharocin	1.541	0.002	Pyrophosphoric Acid	0.433	0.033
5-Phospho-beta-D-ribosylamine	1.540	0.009	4-Phenolsulfonic acid	0.429	0.046
Dehydrocholic acid	1.50	0.013	Geranyl phosphate	0.412	0.043
Tritoqualine	0.634	0.008	Caftaric acid	0.412	0.008
Benzoyl cyanide	0.628	< 0.001	Disulfaton	0.396	0.023
Ocaperidone	0.622	0.012	4-(1,2-Dihydroxy-2-propanyl)-1-methyl-1,2-cyclohexanediol	0.388	0.021
Monomethyl phosphate	0.621	0.002	Hydrogen bromide	0.363	< 0.001
MFCD00056202	0.619	0.019	(2-Hydroxy-2-oxido-1,3,2-dioxaphospholan-4-yl) methyl palmitate	0.316	0.004
Minosaminomycin	0.589	0.037	Chloralodol	0.228	< 0.001
Trametinib	0.557	0.003	Caprylic acid	0.199	0.024
Premithramycin A3’	0.550	0.004	1,3-Nonanediol acetate	0.187	0.039
Alpha,alpha’-trehalose 6-mycolate	0.546	0.005	Retosiban	0.170	< 0.001
1-stearoyl-2-arachidonoyl- sn-glycero-3-phosphoserine	0.521	0.033	3-Hydroxytridecanoic acid	0.155	0.009
Phytosphingosine	0.521	0.016	Difluprednate	0.126	< 0.001
Depe	0.484	0.006	Leukotriene B4	0.075	< 0.001
Azithromycin	0.462	0.001	Pentachlorophenol	0.071	< 0.001
1-Stearoyl-2-docosahexanoyl- sn-glycero-3-phosphocholine	0.461	0.037	3,4,15-Triacetoxy-12,13-epoxytrichothec-9-en-8-yl 3-methylbutanoate	0.055	< 0.001
Benzyl succinate	0.458	0.003	Nobiletin	0.029	< 0.001
Ascidiacyclamide	0.457	0.005			
1,2-Dioleoyl-sn-glycero-3-phospho- N,N-dimethylethanolamine	0.436	0.011			
12-Deoxyoligomycin A	0.433	0.011			
Terminalin	0.420	0.036			
Digitoxin	0.415	0.028			
Uroporphyrinogen IV	0.362	0.009			
Vilazodone	0.361	0.001			
N,N-Bis(2-hydroxyethyl) dodecanamide	0.334	0.002			
1-Eicosyl-2-docosanoyl-sn-glycero-3- phosphocholine	0.307	0.042			
Emblicanin A	0.275	0.007			
Phytolaccoside B	0.221	0.025			

Serum samples collected on 19 d of the 2nd oestrous cycle. Metabolites with VIP > 1, *P*-value < 0.05, fold change ≥ 1.5 or fold change ≤ 0.65 were considered differential metabolites

The effects of DF intake level on concentrations of SCFAs in faeces and colon chyme of gilts are shown in Table 7. The concentrations of acetate, propionate and butyrate in faeces of gilts on 30 d of the experiment were linearly increased by DF intake level (*P* <  0.05 or *P* <  0.01, Table 7). The concentrations of propionate and butyrate in chyme in the colons of gilts at 19 d of the 2nd oestrous cycle were linearly increased by DF intake level (*P* <  0.05, Table 7).

**Table 7:** Effects of DF intake level on the concentrations (μmol/g) of short-chain fatty acids in faeces and colon chyme of gilts

Items	Treatments				*P*-value	
	1.0 DF	1.5 DF	1.75 DF	2.0 DF	Linear	Quadratic
^1^Faeces at 30 d of experiment						
Acetate	40.1 ± 2.6^b^	41.5 ± 2.9^b^	47.6 ± 1.7^ab^	51.4 ± 2.7^a^	0.002	0.231
Propionate	14.2 ± 1.6^b^	15.5 ± 1.4^ab^	19.2 ± 0.8^a^	19.4 ± 1.1^a^	0.003	0.709
Butyrate	8.4 ± 0.8	9.8 ± 1.2	11.4 ± 1.1	12.1 ± 0.7	0.010	0.843
^2^Chyme in colon at 19 d of the 2nd oestrous cycle						
Acetate	58.9 ± 2.9	56.8 ± 4.8	63.7 ± 3.0	62.6 ± 3.0	0.324	0.604
Propionate	25.8 ± 1.1	24.8 ± 1.9	29.8 ± 1.2	31.8 ± 3.5	0.046	0.218
Butyrate	11.6 ± 0.4^b^	12.5 ± 1.3^ab^	13.1 ± 0.8^ab^	15.4 ± 1.0^a^	0.012	0.263

Faeces tested at 30 d of the experiment, *n* = 8; Chyme colon data, *n* = 6; Data are expressed as means ± S.E.; *DF*, dietary fiber; Means with different letters ^a,b^ denote *P* < 0.05

The effects of DF intake level on serotonin concentrations in the serum and follicular fluid in gilts are presented in Table 8. The serum serotonin concentrations on 30 d of the experiment increased linearly with increasing DF intake level (*P* = 0.001, Table 8). The elevation in DF intake level resulted in a linear increase in serotonin in serum (*P* <  0.001, Table 8) and in follicular fluid (*P* = 0.032, Table 8).

**Table 8:** Effects of dietary fiber intake level on serotonin concentration in serum, follicular fluid and colon tissues of gilts

	Treatments				*P*-value	
	1.0 DF	1.5 DF	1.75 DF	2.0 DF	Linear	Quadratic
At 30 d of experiment						
Serum, ng/mL	1422.1 ± 86.6^b^	1713.4 ± 1115.3^ab^	2069.1 ± 133.8^a^	1879.5 ± 105.5^a^	0.001	0.226
FF, pg/mL	209.8 ± 16.0	259.9 ± 24.9	286.1 ± 16.8	304.5 ± 32.1	< 0.001	0.356
At 19 d of the 2rd oestrous cycle						
Serum, ng/mL	646.8 ± 42.4^c^	847.3 ± 32.2^b^	1026.8 ± 68.5^ab^	1141.0 ± 51.9^a^	< 0.001	0.598
FF, pg/mL	112.7 ± 10.0	124.8 ± 27.8	179.6 ± 35.8	180.1 ± 13.1	0.032	0.724
Colons, ng/mg	2.84 ± 0.28^b^	3.05 ± 0.24^b^	4.58 ± 0.20^a^	4.11 ± 0.09^a^	< 0.001	0.928

Data are expressed as means ± S.E.; means with different letters ^a,b^ denote *P* < 0.05. *DF*, dietary fiber; *FF*, follicular fluid; *n* = 6

Melatonin concentrations in follicular fluid increased linearly with increasing DF intake level at 30 d of the experiment and at 19 d of the 2nd oestrous cycle (Supplementary Fig. 3).

Linear regression analysis results between butyrate concentration in colon chyme and serotonin in serum, follicular fluid, and colon tissues are presented in Table 9. A positive linear association was observed between butyrate concentration in colon chyme and serotonin in serum, follicular fluid and colon tissues (*P* <  0.01, Table 9).

**Table 9:** Linear regression between butyric acid (x, μmol/g) and serotonin in different tissues^1^

Items	b_0_	b_1_	R^2^	*P*-value
Serotonin				
Serum, ng/mL	24.705	67.739	0.624	< 0.001
Follicular fluid, pg/mL	−118.494	20.362	0.697	< 0.001
Colon, ng/mg	1.266	0.181	0.281	0.008

^1^The linear regression model is *y* = *b*_*0*_ + *b*_*1*_ × *x*, where b_0_ denotes serum serotonin concentrations when the butyrate concentration in colonic content was 0 μmol/g, and b_1_ denotes the serotonin increment when the colonic butyrate content was increased to 1 μmol/g

## Discussion

DF is an anti-nutritional factor that exerts negative effects on nutrient digestion, and sometimes diminishes growth performance [20]. However, basal diet supplemented with graded amounts of DF from a 33 kg phase did not negatively impact growth performance and the age at puberty in gilts [7]. Oocyte maturation, a parameter reflecting the quality of oocytes, is a determining factor influencing early embryo development [21, 22]. Previous research revealed that the beneficial effects of fiber-rich ingredients on early embryonic survival could be attributed to enhanced oocyte maturation in gilts [5, 6]. Consistently, results from our recent studies demonstrated that DF could improve the survival rate of immature oocytes, thereby improving the ovarian reservation of replacement gilts [7, 13]. The current findings, coupled with the results of a companion study [7], proved beneficial effects of DF on both the number and quality of oocytes in growing gilts. The quality of replacement gilts not only plays an important role in pubertal maturation, but also influences the lifetime fertility of sows [23]. The successful reproductive process of sows requires a continuous supply of mature oocytes and the secretion of reproductive hormones such as oestrogen and progesterone from granulosa cells, which is largely determined by the number and quality of oocytes in ovaries [24–26]. Therefore, DF consumption during the replacement phase may exert a benefit on the lifetime fertility of sows, although this needs further validation.

Interestingly, our results implied that uterine development was also significantly promoted by DF intake level. To date, very few data are available on the nutrient-dependent regulation of the uterus in gilts. Recent evidence found that dietary energy density and lysine level had no effect on uterine development [27]. Weaver et al. observed a 118 g heavier uterine weight on 19 d of the oestrous cycle in gilts fed a fiber-rich diet compared with a low-fiber diet [6]. The development of uterus at mating plays an important role in regulating early embryonic survival, since foetus would die if uterine endometrial lumen epithelium was insufficient to provide support to foetus development [28]. Therefore, replacement gilts fed a high DF diet during their rearing phase could benefit from subsequent improved fertility.

DF consists of nondigestible carbohydrates that are resistant to digestion and absorption in the porcine gastrointestinal tract. Hence their metabolism requires microbiota harboured in the gut. Indeed, microbial metabolism of DF is the key process mediating the beneficial effects of DF on gastrointestinal health and disease resistance [17, 29]. DF significantly alters the gut microbial diversity of hosts, by stimulating the growth of fiber-degrading microbiota, and this alternation in gut microbial diversity in turn impacts gut microbial ecology, host physiology, and health [17, 29]. In order to investigate the role of microbiota in the regulation of oocyte maturation by dietary fiber, we explored microbial diversity by 16S rRNA sequencing. In the present study, the Observed_species, Shannon index, and Chao1 index of DF 1.5 gilts were lower than those of DF 1.75 and DF 2.0 groups, indicating that DF intake level altered the alpha diversity of microbiota in the gut. With increasing DF intake level, the relative abundance of 13 microbiota genera were significantly increased, among which *Lactobacillus*, *Faecalibacterium*, *Prevotella_9*, *Alloprevotella*, *Prevotellaceae_UCG-003*, *Prevotella_7*, *Fibrobacter*, *Sphaerochaeta* and *Erysipelotrichaceae_UCG-004* are able to mobilise DF to produce SCFAs. Studies conducted on zebrafish revealed that the probiotic *Lactobacillus rhamnosus* exerted beneficial effects on oocyte development [30, 31]. Additionally, *Faecalibacterium* was implicated to play a role in the pathogenesis of polycystic ovary syndrome (PCOS) in human [32–34]. On the other hand, 17 microbiota genera significantly decreased with increasing DF intake level, including *Streptococcus* and *Escherichia-Shigella*, which are pathogenic bacteria. A recent study revealed that an elevation in *Bacteroides vulgatus* was an important factor leading to PCOS in human [35]. Furthermore, studies conducted on humans revealed that the diversity of the gut microbiota is closely correlated with the morbidity of PCOS, in particular for those with obesity [34–36]. However, it remains unclear how the microbiota influences ovarian development in domestic animals such as pigs.

We conducted serum metabolomics analysis to explore the potential mechanism mediating the effects of DF on oocytes in gilts. The untargeted metabolomics analysis revealed significant changes in serum metabolites. Compared with gilts in the DF 1.0 group, the total number of metabolites that differed from those in DF 1.5, DF 1.75 and DF 2.0 groups was 92, 123 and 171, respectively, indicating a dose-dependent regulation of DF intake level on serum metabolites. However, oocyte quality reached a peak in DF 1.75 gilts, implying an optimal DF intake level for replacement gilts, but the reason why a further increase in DF intake level resulted in no further improvement in reproductive traits compared with DF 1.75 gilts remains unclear and awaits further investigation.

We further explored the differential metabolites between DF 1.0 and DF 1.75 groups. Interestingly, KEGG pathway analysis revealed that some of those metabolites were gut-derived. For example, the serotonergic pathway derived from tryptophan metabolism in enterochromaffin cells of the gastrointestinal tract, in which tryptophan is converted to serotonin (also known as 5-HT) by the enzyme tryptophan hydroxylase 1 encoded by the *TPH1* gene [37]. It has been revealed that metabolites produced by the gut microbiota, such as SCFAs, bile acids, cholate, deoxycholate and p-aminobenzoate, can up-regulate *TPH1* gene expression and thereby stimulate serotonin secretion [38]. Over 95% of serotonin is gut-derived, and serotonin is believed to be a gut-derived metabolic signal [38, 39]. In order to validate the effect of DF intake level on serotonin secretion, we measured the serum concentration of serotonin, and revealed a linear effect of DF intake on serum serotonin level. Consistently, the serotonin level in follicular fluid was also elevated by DF intake level. Serotonin receptors such as 5-hydroxytryptamine (HTR)1D, 5-HTR2 and 5-HTR7 are expressed in porcine ovarian tissues [13]. Injection of serotonin into crustaceans [40–42] and fish [43] resulted in improved ovarian follicular development and oocyte maturation. Serotonergic signalling in mammalian ovarian follicles and oocytes might play important roles in oocyte or early embryo survival [44]. Deletion of the rate-limiting enzyme-encoding gene *Tph1* resulted in elevated embryo death from 3.6% in wild-type to 80–89% in mice lacking *Tph1* [45]. Therefore, serotonin might be one of the potential regulators mediating the effects of DF on oocyte maturation in gilts. Additionally, serotonin serves as the sole precursor of melatonin via the rate-limiting enzyme arylalkylamine-N-acetyltransferase (AANAT), the expression of which can be up-regulated by the microbial metabolite butyrate in duodenal tissue and Caco-2 cells [46]. To clarify, we observed a dose-dependent effect of DF on the concentration of melatonin in follicular fluids. Several lines of evidence have demonstrated that melatonin can promote the developmental competence of porcine oocytes [47–49]. Thus, the serotonin-melatonin pathway appears to be involved in the control of oocyte maturation following DF intake.

In addition, sphingolipid metabolism differed between gilts in DF 1.0 and DF 1.75 groups. Sphingolipids are lipids with a set of aliphatic amino alcohols that play an important role in cell recognition and signal transduction [50]. Ceramides are early products of sphingolipid synthetic pathways involved in the control of hepatic gluconeogenesis induced by the microbiota-bile acid pathway [51], and they impair porcine oocyte quality via regulation of mitochondrial oxidative stress and apoptosis [52, 53]. Bile acids, steroid acids primarily produced by the liver, are secreted into the gut lumen upon feeding to assist the absorption of nutrients such as lipids and vitamins, glucose homeostasis, and regulation of energy expenditure [54]. Liver-derived bile acids in mammals are usually considered primary acids, and most are re-absorbed via enterohepatic circulation. However, a small fraction of this pool (roughly 5%) is able to escape reabsorption in the ileum and undergoes bacterial transformation in the colon, giving rise to secondary bile acids. In this study, the secondary bile acids deoxycholic acid and taurochenodeoxycholic acid were increased in DF 1.75 gilts compared with DF 1.0 gilts. Tauroursodeoxycholic acid was shown to facilitate DNA damage repair and improve early embryo development in pigs [55] and other mammals [56]. Additionally, DF intake also altered levels of other metabolites, including spermidine [57], 4-aminobenzoic acid [58] and ibuprofen [59] that are known to influence oocyte quality or reproductive function. However, we cannot exclude the possibility that these differentially abundant metabolites might act as primary signals to trigger secondary metabolic signals that influence oocyte and uterine development.

## Conclusion

The current study provides evidence showing that increased DF intake exerts profound beneficial effects on oocyte maturation and uterine development in gilts. Notably, feeding replacement gilts additional intake of 419.5 g/d DF in the form of inulin and cellulose at a 1:4 ratio on a corn-soybean meal based diet could optimize the oocyte and uterine development. We also observed that DF might increase the SCFA-producing microbe and gut-derived metabolites (such as serotonin) to exert the benefit on the oocyte quality and uterine development of replacement gilts, and thereby providing new microbial and metabolomic insight into the mechanisms mediating the effects of DF. The findings could help develop optimal nutritional strategies for replacement gilts, as well as dietary patterns for other mammals, including humans.

## Supplementary Information

**Additional file 1.** Supplementary figures.

## Abbreviations

DF: Dietary fiber
LY: Landrace × Yorkshire
SD: Standard deviation
SCFAs: Short-chain fatty acids
CF: Crude fiber
NDF: Neutral detergent fiber
COC: Cumulus-oocyte complex
ELISA: Enzyme-linked immunosorbent assay
BMP15: Bone morphogenetic protein 15
GDF9: Growth differentiation factor 9
PCA: Principal component analysis
PCOS: Polycystic ovary syndrome
TPH1: Enzyme tryptophan hydroxylase 1
AANAT: Arylalkylamine-N-acetyltransferase
