# Supplementary material for: Microbial and metabolomic mechanisms mediating the effects of dietary inulin and cellulose supplementation on porcine oocyte and uterine development
Source: J Anim Sci Biotechnol. 2022 Jan 13;13:14. doi: 10.1186/s40104-021-00657-0 (PMC8760789; doi:10.1186/s40104-021-00657-0)
Supplement: Supplementary file 1 — Additional file 1. Supplementary figures. [file 40104_2021_657_MOESM1_ESM.pdf]

Supplementary figures

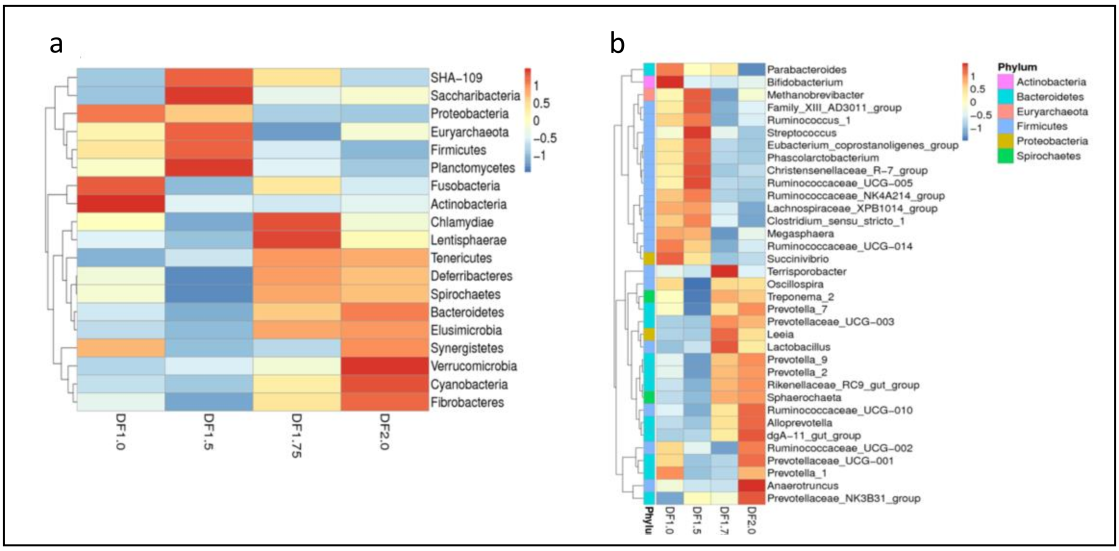

Fig. 1 Heatmap of abundance of the fecal microbiota composition at the phylum level (a) and the genus level (b). Faecal samples were collected from gilts at the 19th of the 2nd estrous cycle. DF, dietary fiber. n = 8 for each treatment.

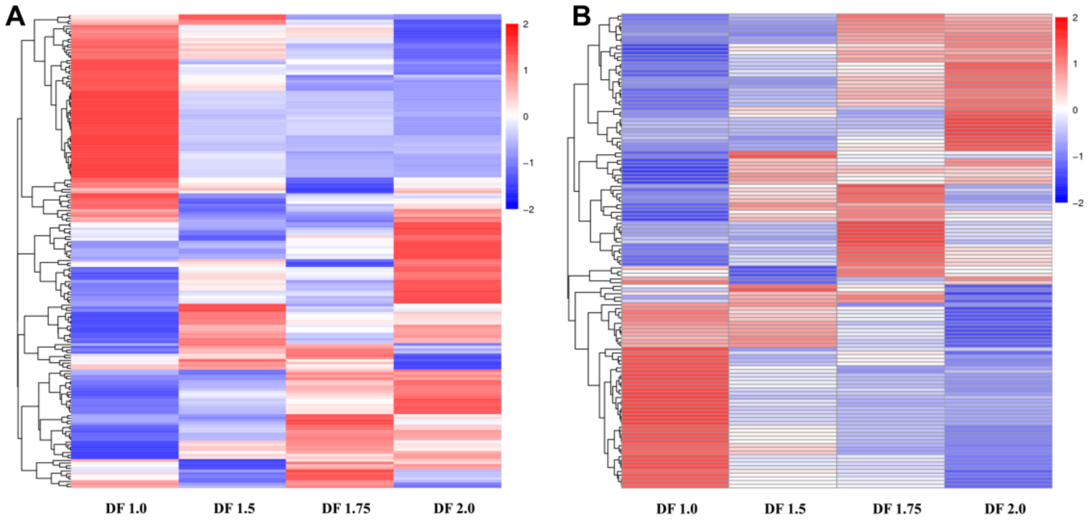

Fig. 1 Differentially altered serum metabolites revealed significant changes in hierarchical clustering identified in positive (a) and negative (b) ionisation modes. Serum samples were collected from gilts at the 19th of the 2nd estrous cycle. DF, dietary fiber. n = 6 for each treatment.

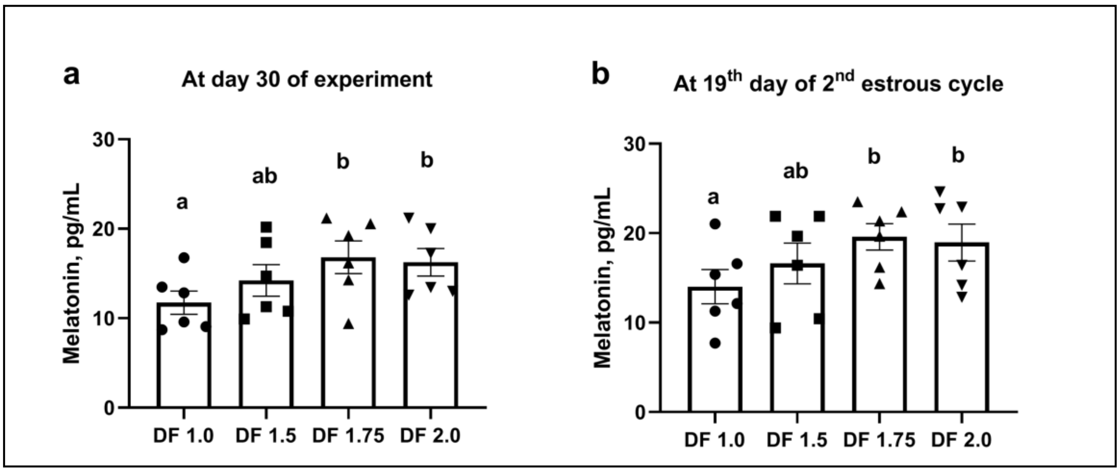

Fig. 3 Effects of DF intake levels on the melatonin concentration in follicular fluid of gilts at day 30 of experiment (a) and at the 19th day of 2nd estrous cycle. Means with different letter [a,b] denotes $P < 0.05$. DF, dietary fiber; $n = 6$ per groups.

Supplementary Tables

Table 1 Numbers of differentially altered metabolites between groups

Compared Samples	Ionization Mode	Num. of Total Ident	Num. of Total Sig.	Num. of Sig. Up	Num. of Sig. down
DF1.0 *vs.* DF1.5	Positive	868	53	34	19
DF1.0 *vs.* DF1.75	Positive	868	66	40	26
DF1.0 *vs.* DF2.0	Positive	868	98	56	42
DF1.5 *vs.* DF1.75	Positive	868	27	10	17
DF1.5 *vs.* DF2.0	Positive	868	53	24	29
DF1.75 *vs.* DF2.0	Positive	868	32	16	16
DF1.0 *vs.* DF1.5	Negative	462	39	13	26
DF1.0 *vs.* DF1.75	Negative	462	57	20	37
DF1.0 *vs.* DF2.0	Negative	462	73	41	32
DF1.5 *vs.* DF1.75	Negative	462	18	3	15
DF1.5 *vs.* DF2.0	Negative	462	50	25	25
DF1.75 *vs.* DF2.0	Negative	462	18	12	6

Num. of Total Ident, number of total identified metabolites; Num. of Total Sig., number of total metabolites that were statistically different; Num. of Sig. Up, number of total metabolites that were significantly upregulated; Num. of Sig. down, number of total metabolites that were significantly downregulated.

Supplemental methods

Microbial analysis

Total bacterial DNA of feces samples were extracted using the MO BIO PowerFecal® DNA Isolation Kit (Catalog No. 12830-50, MO BIO Laboratories, Inc) according to the manufacturer's protocols. Before sequencing, the concentration and purity of the extracted genomic DNA were measured. The integrity of the extracted genomic DNA was determined by electrophoresis on a 1% (w/v) agarose gel. The concentration and purity of the extracted genomic DNA were measured. According to the concentration, DNA was diluted to1ng/μL using sterile water. The DNA samples were sent to Novogene Bioinformatics Technology (Beijing, China) to perform amplicon pyrosequencing on the Illumina HiSeq PE250 platforms. The V4 hypervariable region of the 16S rRNA gene was amplified using 515F and 806R primer (5′-GTGCCAGCMGCCGCGGTAA-3′ and 5′-GGACTACHVGGGTWTCTAAT-3′, respectively). Raw Paired-end reads obtained by Illumina HiSeq sequencing were spliced. The splicing sequences were called raw tags. Quality filtering on the raw tags were performed under specific filtering conditions to obtain the high-quality clean tags (Bokulich et al., 2013) according to the QIIME(V1.7.0, http://qiime.org/index.html) (Caporaso et al., 2010) quality controlled process. And then chimeric filtering to get Effective Tags as shown in Supplemental Table S3. The effective tags were mapped to OTUs using Uparse software (v7.0.1001 http://drive5.com/uparse/) at 97% sequence similarity. Representative sequence for each OUT was screened for further annotation. The Ribosomal Database Project (RDP) classifier Version 2.2 was used to assign a taxonomic rank to each representative sequence. OTUs abundance information was normalized using a standard of sequence number corresponding to the sample with the least sequences. Subsequent analysis of alpha diversity and beta diversity were all performed basing on this output normalized data. The relative abundance of each OTU was examined at different taxonomic levels. At the phylum level, as the sum of the top 10 phyla with relative abundance exceeded 98%, we selected the top 10 phyla for statistical analysis

with the control group as the reference. At the genus level, we selected genera with relative abundance of more than 0.1% in at least one sample for statistical analysis.

Untargeted Metabolomics

Metabolites extraction

The serum samples 100 μL were added with 80% methanol (400 μL) prechilled on ice followed by well vortexing, incubating on ice for 5 min, and centrifuged at 15000 rpm at 4℃ for 5 min. Supernatants were diluted with LC-MS grade water to final concentration containing 60% methanol, and then were transferred to a fresh Eppendorf tube with 0.22 μm membrane filter. Then they were centrifuged at 15000 g at 4 ℃ for 10 min to obtain the injecting samples.

UHPLC-MS/MS analysis

The untargeted metabolomics was analyzed with LC-MS/MS using a Vanquish UHPLC system (Thermo Fisher) coupled with an Orbitrap Q Exactive series mass spectrometer (Thermo Fisher). The filtrate samples were injected onto an Hyperil Gold column (100 × 2.1 mm, 1.9 μm) using a 16-min linear gradient at a flow rate of 0.2 mL/min. The 0.1% formic acid in water (eluent A) and methanol (eluent B) were used in the positive polarity mode, and negative polarity mode were 5 mM ammonium acetate (pH 9.0, eluent A) and methanol (eluent B). The solvent gradient was 2% B (1.5 min), 2-100% B (12.0 min), 100% B (14.0 min), 100-2% B (14.1 min), 2% B (16 min). Q Exactive mass spectrometer was operated in positive/negative polarity mode with spray voltage of 3.2 kV, capillary temperature of 320 ℃, sheath gas flow rate of 35 arb and aux gasflow rate of 10 arb.

UHPLC-MS/MS analysis

The raw data files generated by UHPLC-MS/MS were processed using the Compound Discoverer 3.0 (CD 3.0, Thermo Fisher) to perform peak alignment, peak picking, and quantitation for each metabolite. The main parameters were set as follows: retention time tolerance, 0.2 minutes; actual mass tolerance, 5ppm; signal intensity tolerance, 30%; signal/noise ratio, 3; and minimum intensity, 100000. After that, peak intensities were normalized to the total spectral intensity. The normalized data was used to predict the molecular formula based on additive ions, molecular ion peaks and

fragment ions. And then peaks were matched with the mzCloud (https://www.mzcloud.org/) and ChemSpider (http://www.chemspider.com/) database to obtained the accurate qualitative and relative quantitative results. Statistical analyses were performed using the statistical software R (R version R-3.4.3), Python (Python 2.7.6 version) and CentOS (CentOS release 6.6), when data were not normally distributed, normal transformations were attempted using of area normalization method.

Data Analysis

These metabolites were annotated using the KEGG database (http://www.genome.jp/kegg/), HMDB database (http://www.hmdb.ca/) and Lipidmaps database (http://www.lipidmaps.org/). Principal components analysis (PCA) and Partial least squares discriminant analysis (PLS-DA) were performed at metaX (a flexible and comprehensive software for processing metabolomics data). We applied univariate analysis (t-test) to calculate the statistical significance (P-value). The metabolites with VIP > 1 and P value < 0.05 and fold change ≥ 1.5 or fold change (FC) ≤ 0.65 were considered to be differential metabolites. Volcano plots were used to filter metabolites of interest which based on Log2 (FC) and -log10 (P-value) of metabolites. The data were normalized using z-scores of the intensity areas of differential metabolites and were ploted by Pheatmap package in R language for clustering heat maps. Statistically significant of correlation between differential metabolites were calculated by cor.mtest in R language. P value < 0.05 was considered as statistically significant. The functions of these metabolites and metabolic pathways were studied using the KEGG database. The metabolic pathway enrichment of differential metabolites were performed, when ratio were satisfied by x/n > y/N, metabolic pathway were considered as enrichment, when P value of metabolic pathway < 0.05, metabolic pathway were considered as statistically significant enrichment.
